# To explore the impact of augmented reality digital picture books in environmental education courses on environmental attitudes and environmental behaviors of children from different cultures

**DOI:** 10.3389/fpsyg.2022.1063659

**Published:** 2022-12-07

**Authors:** Shih-Yeh Chen

**Affiliations:** National Taitung University, Taitung, Taiwan

**Keywords:** AR digital picture book, science learning self-efficacy, cross-cultural environmental education, environmental attitudes, environmental behavior

## Abstract

**Introduction:**

Environmental education has long been closely related to sustainable development. In this study, in response to the United Nations Sustainable Development Goals (SDGs), an augmented reality (AR) digital picture book was created using the unique natural ecosystem of Taiwan’s Orchid Island as a source of self-efficacy for science learning.

**Methods:**

Interactive environmental education learning through AR drawing was used to determine whether students’ science learning self-efficacy and environmental attitudes significantly influenced the environmental behavioral skills of culturally diverse children. In this study, 26 elementary sixth-grade Taiwanese students and 26 elementary sixth-grade Japanese students were invited to participate in an extended reality drawing activity as an environmental education curriculum.

**Results:**

Based on the sample size of 52, the survey results were accurate with a sampling error of 3.8% with a confidence level of 95%. A questionnaire survey was administered to the 52 students after the event. After the valid questionnaire samples were collected, a partial least squares structural equation modeling (PLS-SEM) analysis was conducted with Smart PLS 3.0 on the small sample. The results of the study showed that students who had a better self-efficacy in science learning were more likely to engage in conservation actions related to the natural environment in their daily lives.

**Discussion:**

In this study, the constructs of environmental behavior were further discussed and the hypothesis model was validated using the quantitative empirical method. The results of the study revealed good reliability, convergent validity, and discriminant validity of the constructs in the hypothesis model, and the hypothesis model itself was validated. In the validated model, students’ science learning self-efficacy affects the sustainability of their environmental behaviors, but only through the role of environmental attitudes. However, the environmental attitudes construct plays a fully mediating role in the model.

## Introduction

Since people’s understanding of the environment is incomplete and ever-changing, one of the ongoing studies that needs to be conducted is the study of educational development (Gough, 2009). Education is one of the most important assets that enables a person to compare and learn from the positive and negative aspects of life in order to make better decisions for forming a better future (Beier and Rittmayer, 2008). However, there are many issues in educational development that can be explored, and one of them is the research on students’ behavior. Jurigová and Tucková (2016) argue that education can influence the behavior of students, which makes it possible for well-educated students to have a positive impact on the socio-economy, which leads to the innovation occurrences. However, every innovation is not easily accepted and implemented (Sugandin et al., 2018), and the innovation process may not only waste natural resources, but also pollute the environment (Steffen et al., 2015; Barnosky and Hadly, 2016). In light of this, UNESCO proposed the 2030 Action Plan for Education in 2015, which provides country-specific implementation guidelines for Sustainable Development Goals (SDGs) (UNESCO, 2015a,b, 2016). Among the expectations and challenges for quality learning, teachers are highlighted as the key to enhancing quality learning, and a variety of organizations (including profit-making businesses, civic groups, social enterprises, etc.) are expected to participate in educational innovation and investment (Naidoo, 2017). By now, much of the discussion about education aid and development policies in the international community has returned to a review of “making learning happen” (World Bank, 2018). Therefore, environmental education is becoming more and more important, especially for students to learn how to control their behavior toward the environment (Tan and Lau, 2009; Emanuel and Adams, 2011). The aim of environmental education is not only what children should learn about the environment, but also to build children’s correct attitudes and positive relationships toward the environment, so designing the appropriate curriculum is important to achieve the learning goals (Surjanti et al., 2018). However, self-efficacy has an important influence on human motivation and also affects how people feel and think (Bandura, 1977). This makes people with low self-efficacy tend to think that the task or problem they are facing seems more difficult than it really is. In contrast, strong thoughts about self-efficacy produce a sense of calm and challenge when faced with difficult tasks (Clark and Button, 2011). Hence, De Oliveira and Rodrigues (2010) stated that participatory ecological learning helps develop sustainable behaviors to accelerate the achievement of SDGs, and Pane and Patriana (2016) found that over 90% of students were willing to engage in environment-based learning.

This study aims to explore the issue of eco-learning from an educational perspective, especially in relation to sustainability programs (Pellas, 2014; Huang, 2016; Gould et al., 2018). As highly contextualized carriers, picture books can be better used for learning through storytelling with pictures (Damayanti and Febrianti, 2020). Different from traditional picture books, AR picture books can present many subjects in an interactive way, such as environmental conservation (Kamarainen et al., 2013), earth science (Wei et al., 2015), programming (Lin and Chen, 2020), art and culture, etc. (tom Dieck and Jung, 2017). AR technology can facilitate collaboration among students and increase their motivation to learn (Radu, 2014). For students, interactive AR displays through image recognition and graphic rendering processes add dimensions to the learning of digital picture books. Therefore, interactive learning environments often have a positive impact on student learning (Chen and Wang, 2015; Ho et al., 2017), possibly due to the novelty of the experience, the opportunity to be welcomed by other students, and the fact that teachers allow students to move around the classroom (Godwin-Jones, 2016). Through visual and auditory experiences that are different from traditional teaching, teaching in AR scenarios facilitates teachers to convey knowledge and effectively receive feedback from students (Soltani and Morice, 2020).

Therefore, this study aims to develop digital picture books by integrating local Taiwanese culture through AR development platform. Based on science learning self-efficacy, the augmented reality (AR) digital picture book created with the unique natural ecosystem of Taiwan’s Orchid Island was used to test students’ perceptions of environmental education and sustainable behavior from different cultures.

## Literature review

Environmental education is a conservation strategy that provides opportunities for collaborative work with scientists, policymakers, community members, and other stakeholders. Thus, environmental education is often based on local contexts to highlight local knowledge, cultural experiences, values, and practices, and in this way, it encourages productive interactions among many groups, including those that may be marginalized (Toomey et al., 2017). By definition, environmental education includes developing and supporting environment-related attitudes, values, awareness, knowledge, and skills that prepare people to take informed action for the environment (UNESCO, 1978; Monroe et al., 2019). Conducting relevant high quality scientific research and sharing the findings with decision makers to address complex environmental protection issues (Lemos et al., 2018; Knight et al., 2019). Therefore, the research often focuses on results at different scales, including the individual level (e.g., individual environmental attitudes or behaviors), the societal level (e.g., community capacity building), and the ecosystem level (e.g., populations of endangered species). Based on a growing body of research highlighting the complexities of behavior, environmental education is no longer a linear pathway from developing attitudes toward the environment to learning about protecting it and then to taking action to improve it, and now emphasizes the dynamic, complex relational ecosystems that influence behavior (Marcinkowski and Reid, 2019). Taking advantage of the cross-disciplinary properties of the field of environmental research, environmental educators incorporate principles from behavioral psychology, health education, marketing, learning sciences, and sociology, and this diversity of perspectives and theoretical frameworks guides what researchers envision as effective practices in the field (Heimlich and Ardoin, 2008; Jacobson et al., 2015). These practices include having place-based experiences to be part of a community that sets common social and environmental norms, to understand and build connections to the local environment, to build and hone action-related skills, and to act on meaningful issues (Monroe and Krasny, 2016; Niemiec et al., 2016). There are many steps required to actually improve the environment in a way that involves identifying and specifying environmental education in order to be able to evoke actions and behaviors for environmental protection, such as climate change and biodiversity loss. Thus, most of these issues are tied to the inherent complexity of social-ecological systems (Toomey et al., 2017; Knight et al., 2019). Nevertheless, understanding how environmental education programs are successful and then contextualized often involves how to measure short- and medium-term outcomes (e.g., environmental issues, self-efficacy, critical thinking) and consistently track those that take more time to develop and demonstrate (Ardoin et al., 2015). In general, high quality environmental education usually involves many partners and stakeholders who collaborate in science, decision making, and research implementation spaces at the intersection of local culture and environment, which makes it important for environmental education evaluations to consider these productive but complex implementation spaces (Toomey et al., 2017). Ecotourism as an environmentally friendly approach to educational tourism activities has a variety of concerns and meanings and is known as green tourism (Furqan et al., 2010; Funck and d’Hauteserre, 2016), but some places worthy of ecotourism are difficult to reach, and Reading can help people experience those difficult places (Caesar, 2018). For children, it is an immersive, full-sensory experience in which social interaction and symbolic mediation are two intrinsically relevant factors that contribute to the individual development of key cognitive skills (Vygotsky, 1978). Storytelling is important for all ages and cultures and can use different forms such as oral storytelling, painting, written text, drama, television, virtual reality (VR) and AR (Gröppel-Wegener and Kidd, 2019). In particular, immersive storytelling content uses virtually generated places, characters, and objects that can be informative, emotional, and memorable, appealing to a variety of audiences (Azuma, 2015), which makes the application of AR in education significant, not only for making mobile learning more possible, but also for providing students with different learning experiences in digital learning environments (Arici et al., 2019).

In recent years, AR has become a hot topic in the field of education (Ibáñez and Delgado-Kloos, 2018), and its promotion in education may come from its media properties such as sensory immersion, navigation, and manipulation (Cheng and Tsai, 2013). With the development of AR technology, researchers have started to apply it to digital picture books (Kim, 2017; ChanLin, 2018; Suryani et al., 2021), which are stories that are digitized with images, text, multimedia audio and video, and interactive features (Rubegni et al., 2021). According to multimedia cognitive learning theory (Mayer, 2003), deeper learning occurs when messages are presented in both verbal and non-verbal formats, so this study of digital graphics includes the presentation of both verbal and non-verbal messages. The dual coding theory (Paivio, 2007) also suggests that verbal and non-verbal information is processed in two mutually exclusive channels. Therefore, digital picture books enhance children’s comprehension of stories and vocabulary more than traditional picture books (Takacs et al., 2015; Takacs and Bus, 2016), and AR picture books with multimedia content not only promote students’ learning and understanding, but also inspire children’s imagination and improve their motivation to learn (Lin et al., 2018; Danaei et al., 2020). AR technology can superimpose digital images on real environments to represent different worlds and make virtual objects appear to be part of the real world (Furht, 2011; Alyousify and Mstafa, 2022). Therefore, AR technology has two important properties, namely the ability to register objects in a virtual three-dimensional space and the ability to interact with each terminal device in real time (Azuma, 1997). In other words, virtual objects are added to the real environment in real time during the user experience (Cipresso et al., 2018), which allows learners with limited computer experience to create unique educational advantages and new learning possibilities through the seamless perception between virtual and physical elements of AR technology (McKenzie and Darnell, 2004). By interacting with synthetic audiovisual content, AR technology can enhance children’s understanding of book content (Dias, 2009). Therefore, AR technology must include three functions: tracking real-world objects, information processing, and presenting information synthetically to the user (Carmigniani et al., 2011). Handheld devices are one of the most common ways to implement AR technology, allowing users to have a new sensory experience by overlaying virtual objects in a real-world environment (Giunta et al., 2018). Some studies have shown that digital picture books can enhance the learning effect, while others have shown that digital picture books are not different from traditional picture books. At the same time, many researches have shown that the design of multimedia elements in picture books may increase the cognitive load of readers and distract them from learning (Takacs et al., 2015). Teachers must improve their teaching abilities and skills because their proficiency significantly affects the teaching process and students’ mastery of the curriculum, so the selection of appropriate learning methods and content media for students becomes a factor that can successfully improve students’ learning effects (Surjanti and Soejoto, 2018). If AR technology can be properly used in teaching, it can be beneficial in solving educational problems such as lack of classroom time, crowded classrooms, and inexperienced teachers (Akçayır and Akçayır, 2017). Regarding environmental curriculum issues, students are taught about the relationship between human behavior and the social environment, and how humans affect natural resources (Clark and Button, 2011). In contrast to previous studies, the lack of environmental awareness among students has led to a plethora of educational and environmental issues, even with economic and social implications (Emanuel and Adams, 2011; Pellas, 2014), while some studies suggest that Education for Sustainable Development (ESD) can solve this problem, ESD can address this issue (Gould et al., 2018). Learning through AR technology can help increase students’ motivation, satisfaction, attention, engagement, and enjoyment (Parmaxi and Demetriou, 2020), and these characteristics can not only foster positive emotions in students but also serve as a source of self-efficacy to help them effectively achieve their learning goals (Wu et al., 2013). Beier and Rittmayer (2008) defined self-efficacy as behaviors that influence self-perceptions. Marry (2014) went further and argued that the concept of academic self-efficacy includes academic achievement and performance in addition to prior interests, and that the concept of academic self-efficacy has more incremental predictive value than traditional intelligence in terms of academic achievement (Kornilova et al., 2009). Therefore, considering previous researches, this study hypothesized that self-efficacy can influence learning outcomes and sustainable behaviors. In this study, a modified version of The Sources of Science Learning Self-Efficacy (SSLSE) scale based on Lin and Tsai (2018) was used to explore the impact of using AR environmental education digital picture book learning to develop and improve the environmental attitudes and behaviors of students from different cultural backgrounds.

## Research hypothesis

**FIGURE 1:**
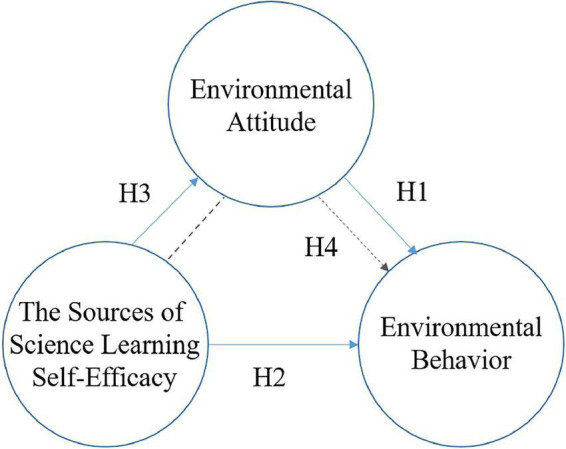
The proposed hypothesized model.

Based on the modified framework proposed by Kaiser et al. (1999), this study incorporated science learning self-efficacy to investigate the effect of science learning self-efficacy on environmental attitudes and environmental behaviors. If environmental attitudes increase, it may positively affect learners’ personal environmental behaviors. Finally, environmental attitudes may play a mediating role between science learning self-efficacy and environmental behaviors, meaning that personal science learning self-efficacy may influence environmental behaviors through changes in environmental attitudes. In view of the above discussion, the four hypotheses are proposed as follows, and the structure of the overall research model is shown in Figure 1.

   (H1): Environmental attitudes positively influence environmental behaviors.

   (H2): Science learning self-efficacy positively influence environmental behavior.

   (H3): Science learning self-efficacy positively affects environmental attitudes.

   (H4): Environmental attitudes play a mediating role between science learning self-efficacy and environmental behaviors.

## Teaching materials and methods

### Research subjects and digital picture books

**FIGURE 2:**
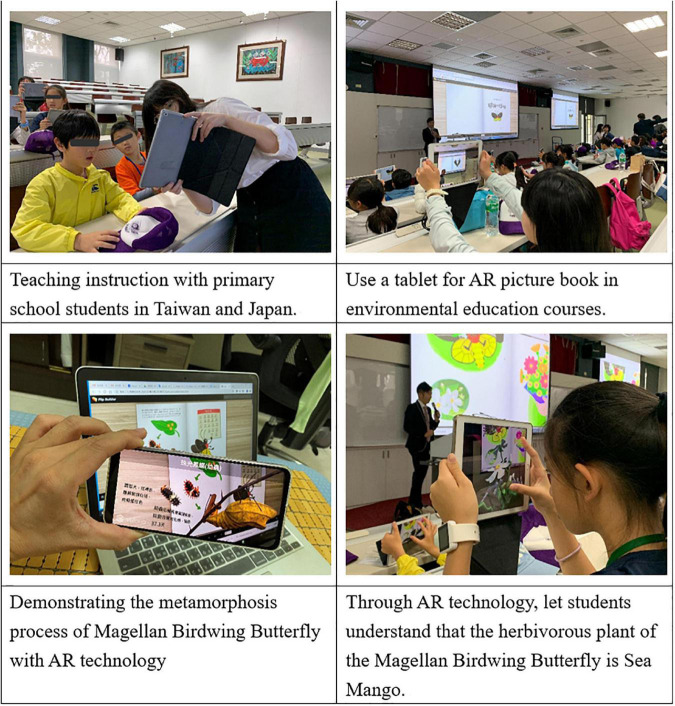
Using AR digital picture books in environmental education courses for children of different cultures.

The subjects of this study were 26 6th grade students from a national elementary school in Taitung, Taiwan and 26 6th grade students from a national elementary school in Kyoto, Japan. Through the partner schools program, teachers and students of the two schools are provided with more opportunities to interact with each other and to support students to connect with the international community, allowing students of the two schools to experience different lifestyles and learning styles. The content of the book follows the natural science curriculum for K-12 education, and the unique species of butterfly “Magellan Birdwing Butterfly” and the unique human ecological environment are used as the background for the story. In addition to the Chinese version of the books, teachers with Japanese language certification N1 were asked to help translate the books into Japanese in order to reduce the learning load of the Japanese students. Finally, for the questionnaire survey, all students were invited to fill out the survey at the end of the course (shown in Figure 2). In this course, 52 valid questionnaires were collected. At the same time, 5 Taiwanese and 5 Japanese students were randomly selected for qualitative interviews. The purpose of the interviews was to understand the students’ opinions, feelings, and suggestions on the use of AR digital picture books in the environmental education curriculum for future teaching improvement.

**FIGURE 3:**
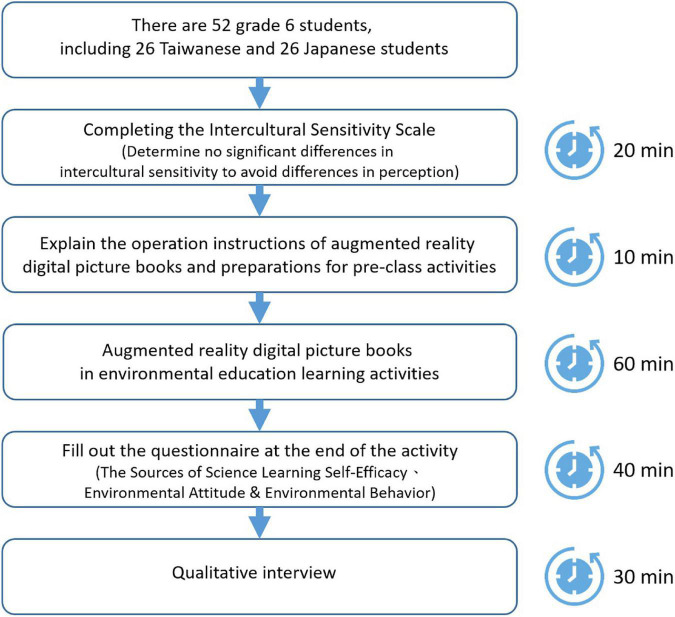
Experiment flow chart of AR picture books.

To improve the intrinsic validity of this study, it was necessary to control the effects of variables that were not related to the study. Therefore, the learning activities were led by the same teacher to avoid the influence of different teaching styles on the study results. In the 20 min before the activity starts, the teacher will ask the students to fill out the cross-cultural sensitivity scale for checking whether the students with different cultural backgrounds have different cultural sensitivity to avoid cognitive differences. After that, the teacher spent 10 min explaining the operation of the AR digital sketchbook and preparing for the pre-school activity. The following 60 min were for environmental education learning activities. After the learning activities, students were asked to spend 40 min to fill out the Science Learning Self-Efficacy Source Scale, the Environmental Attitude Scale, and the Environmental Behavior Scale. Finally, the teachers conducted a 15-min qualitative interview with each of the elementary schools in Taiwan and Japan (shown in Figure 3).

### Scale development

The scale was designed using a structured questionnaire with research hypotheses, and the contents of the questionnaire were divided into science learning self-efficacy sources, environmental attitudes, and environmental behaviors. A seven-point scale was used for the science learning self-efficacy and environmental behaviors section, where people were given a score of 1 to 7 on the extent to which they engaged in the behavior or feeling, representing “almost never,” “rarely,” “occasionally,” “sometimes,” “often,” “usually,” and “almost always.” Regarding the environmental attitude questionnaire, the questions were scored on a seven-point Likert scale, with students being given a score of 1 to 7 on a scale of “strongly disagree,” “disagree,” “slightly disagree,” “average,” “slightly agree,” “agree,” and “strongly agree.”

#### Science learning self-efficacy

The questionnaire questions were based on a modified version of the science learning self-efficacy source scale constructed by Lin and Tsai (2018). The main purpose of Lin and Tsai’s (2018) study was to identify the sources of Taiwanese students’ self-efficacy in science learning, which were subdivided into four relevant dimensions. First, Mastery Experience is the extent to which students interpret previous AR digital picture book learning accomplishments or experienced AR digital picture book events, such as my ability to understand the most difficult aspects of learning with AR digital picture books in a learning environmental education. Second, Vicarious Experience is the extent to which students are exposed to their peers in using AR digital books. These behavioral competencies demonstrate scientific ability, such as when seeing how another student solves a problem with AR digital books for learning environment education, I can see myself solving the problem in the same way. Third, Social Persuasion is the message students receive from peers, parents, teachers, and other adults that encourages their academic abilities, e.g., I feel confident when my parents tell me I am doing a good job of learning environmental education with AR digital picture books. Lastly, Physiological and Affective States (PES) are the extent to which students experience physiological and psychological states such as tension, depression, or anxiety related to science learning, e.g., I feel stress and tension when conducting environmental education lessons with AR digital picture books. In addition, in order to simplify the research model, we selected questions that were more compatible with Taiwanese and Japanese culture and the reading comprehension ability of elementary school students, and reduced the number of questions measured as much as possible. Finally, the four aspects of science learning self-efficacy were three questions on mastery experience, four questions on alternative experience, three questions on verbal persuasion, and three questions on physical and emotional states, for a total of 13 questions.

#### Environmental attitudes

Kaiser et al. (1999) concluded that general environmental attitudes can be discussed in three parts, which include environmental knowledge, environmental values and environmental behavioral intentions and collected 3,000 samples and constructed an environmental attitude scale with 28 total questions. In this study, a modified version of the above scale was used, considering both the environmental geography of Taiwan’s eastern Orchid Island and issues related to environmental education curriculum using AR digital picture books. In addition, the number of questions was reduced to make the model more concise. The total number of questions in this component was divided into 11 questions in three directions: 4 questions on environmental knowledge, 4 questions on environmental values, and 3 questions on environmental behavioral intentions. The environmental knowledge question was about the level of awareness of the natural ecology on the island, such as the reduction in the number of species that might interrupt the food chain and then affect some of the subsequent species in the food chain. Environmental values are values about the natural environment on Lantau Island, such as the right of all living things to exist, whether aquatic, plant, animal or human. Environmental behaviors are intended to be the level of willingness to engage in environmental conservation behaviors, such as preserving the environment in nature reserves and supporting increased environmental conservation fees.

#### Environmental behavior

Based on Smith-Sebasto and D’Costa’s (1995) six dimensions of responsible environmental behaviors, Cheng et al. (2013) refined them to two, General Behavior and Particular Behavior, with four questions each. General behaviors were measured by whether the interviewee would learn to understand and discuss environmental issues with others, such as reading local environmental reports or books after the interview. Particular behaviors were measured to determine whether the respondents would engage in environmental protection actions in their daily lives, such as picking up trash and tree branches when seeing them on the beach.

### Data analysis method

In this study, a narrative statistical analysis was first conducted on the sample characteristics to understand the cross-cultural sensitivity of the students. In addition, since the sample size was about 50 students, it was a small sample analysis. Hair et al. (2019) indicated that partial least squares (PLS) is suitable for model analysis of small samples, and therefore the first three hypotheses will be examined using the statistical software Smart PLS 3.0 for subsequent analysis. In this study, the PLS Algorithm was used to calculate the path coefficients between the models, and the significance of the models was checked by the “Bootstrap” method. Finally, the mediation effect of hypothesis 4 was examined by the method proposed by Baron and Kenny (1986), and the mediation effect was re-examined by the sobel test.

## Results and discussion

### Sample characteristics

As mentioned above, a total of 52 valid samples were taken from 26 6th grade elementary school students in a national elementary school in Taitung, Taiwan and 26 6th grade elementary school students in Kyoto, Japan, of which 28 were male and 24 were female. Based on the sample size of 52, the survey results were accurate within a sampling error of 3.8% with a confidence level of 95%. The Intercultural Sensitivity Scale (ISS) proposed by Chen and Starosta (2000) was used to determine whether there were differences in cultural sensitivity among subjects with different cultural backgrounds. Therefore, one-way analysis of variance was conducted using SPSS, where Interaction Engagement (*F* = 1.87, *p* > 0.05), Respect for Cultural Differences (*F* = 0.61, *p* > 0.05), Interaction Confidence (*F* = 0.86, *p* > 0.05) Interaction Enjoyment (*F* = 0.88, *p* > 0.05), Interaction Attentiveness (*F* = 0.51, *p* > 0.05), confirming that there was no significant difference between subjects. The Cronbach’s alpha values for the SSLSE, environmental attitude, environmental behavior were 0.92, 09.5, and 0.90, respectively. That means the research instrument had a satisfactory quality to evaluate the items.

### Descriptive statistical analysis

#### Science learning self-efficacy

Compared to the environmental attitude level, the students’ learning of environmental education with AR digital picture books was less consistent. Because the experience of using emerging technologies varies from person to person, the overall average is lower than that of environmental attitudes. The highest mean was 5.92 for the questions “ME1: I can do well on tests using AR digital picture books for environmental education learning” and “ME2: I can do well on assignments using AR digital picture books for environmental education learning.” And the next highest mean was 5.85 for the question “VE2: My favorite teacher is usually my natural science teacher” and “SP2: My classmates say I understand everything that is taught in class.” Finally, the lowest mean score was 5.39 for the question “VE4: I feel confident when other students in my class are doing well in the educational learning environment with AR digital books.” According to the aforementioned analysis, it can be found that science learning self-efficacy varies depending on the ability of the teacher and the learning load caused by the content of AR digital picture books.

#### Environmental attitudes

In order to understand the environmental attitudes of the students after learning environmental education, the questionnaire data was analyzed by descriptive statistics, as shown in Table 1. The environmental attitudes can be divided into three aspects: environmental knowledge, environmental values, and environmental behavioral intentions. For the overall question on environmental attitudes, “EV1: All living things, whether aquatic organisms, plants, animals, and humans, have the right to exist” had the highest mean score of 5.73, while “EK3: If carbon dioxide continues to be emitted into the atmosphere in large quantities, the climate may change dramatically” had the next highest mean score of 5.71. The lowest mean score was “EBI2: I am willing to pay environmental tax (e.g., environmental conservation fee),” which was 5.21 points. From the descriptive statistics, students from different cultural backgrounds think that plants and animals have the same right to live as humans, and they also know that the greenhouse effect is the main cause of climate change. However, students do not have the financial means to make their own decisions about paying taxes, so this item is the lowest item in the entire questionnaire.

**TABLE 1:** Descriptive statistics of items for sources of science learning self-efficacy, environmental attitude, and environmental behavior constructs.

Factors and items	M	SD	Factor loading	Cronbach’s α
The sources of science learning self-efficacy (SSLSE)				0.92
**Mastery experience**				
ME1: I can do well on tests using AR digital picture books for environmental education learning.	5.92	1.20	0.84	
ME2: I can do well on assignments using AR digital picture books for environmental education learning.	5.92	1.15	0.85	
ME3: I can understand the most difficult aspects of environmental education learning with AR digital picture books.	5.56	1.30	0.75	
**Vicarious experience**				
VE1: When I see how my natural science teacher solves problems, I can imagine myself solving them in the same way.	5.62	1.28	0.70	
VE2: My favorite teacher is usually my natural science teacher.	5.85	1.29	0.76	
VE3: When I saw how another student solved the problem of using AR digital picture books for learning environment education learning, I could see myself solving the problem in the same way.	5.75	1.19	0.70	
VE4: I feel confident when other students in my class are doing well in the educational learning environment with AR digital books.	5.39	1.27	0.70	
**Social persuasion**				
SP1: I am commended for my ability to use AR digital picture books for learning environment education learning.	5.73	1.29	0.75	
SP2: My classmates say I understand everything that is taught in class.	5.85	1.33	0.64	
SP3: I feel confident when my parents tell me I’m doing a great job of teaching learning in a learning environment with AR digital picture books.	5.62	1.44	0.73	
**Physiological and affective states**				
PAS1: I feel stressed and nervous about environmental education courses with AR digital books.	5.67	1.37	0.66	
PAS2: When I use AR digital picture books for learning environment education, my mind is blank and I can’t think clearly.	5.44	1.38	0.69	
PAS3: I get depressed when I think about using AR digital picture books for learning environment education.	5.77	1.22	0.62	
Environmental attitude				0.95
**Environmental knowledge**				
EK1: Melting polar ice caps can cause coastal and island flooding.	5.67	1.57	0.92	
EK2: All living things (microorganisms, plants, animals, and humans) are interdependent on each other.	5.46	1.51	0.74	
EK3: If carbon dioxide continues to be emitted into the atmosphere in large quantities, the climate may change dramatically.	5.71	1.54	0.84	
EK4: A reduction in the number of species may interrupt the food chain and affect some subsequent species in the food chain.	5.58	1.36	0.86	
**Environmental values**				
EV1: All living things, whether aquatic organisms, plants, animals, and humans, have the right to exist.	5.73	1.22	0.72	
EV2: Animals should have legal rights.	5.44	1.32	0.81	
EV3: The lives of all living things are valuable and worth preserving.	5.58	1.50	0.82	
EV4: The value of the earth is not in people, but in the earth itself.	5.56	1.20	0.79	
**Environmental behavior intention**				
EBI1: To protect the environment of nature reserves, I support raising environmental conservation fees.	5.69	1.45	0.71	
EBI2: I am willing to pay environmental tax (e.g., environmental conservation fee).	5.21	1.43	0.69	
EBI3: I support the ban on cars in environmentally sensitive areas.	5.69	1.54	0.65	
Environmental behavior				0.90
**General behavior**				
GB1: I will try to learn to solve local environmental problems.	5.60	1.11	0.69	
GB2: I will read reports or books about the local environment.	5.79	1.16	0.77	
GB3: I will talk to others about local environmental protection issues.	5.56	1.09	0.71	
GB4: I will try to convince my friends to protect the local natural environment.	5.62	1.14	0.85	
**Particular behavior**				
PB1: When I see others causing damage to the local environment, I will bring it to the attention of the relevant authorities.	5.52	1.02	0.78	
PB2: I will follow the legal channels to stop the damage to the local environment.	5.58	1.37	0.70	
PB3: I will pick up trash and branches when I see them on the beach.	5.67	1.13	0.82	
PB4: If there is a beach cleanup event, I would like to participate.	5.60	1.18	0.73	

#### Environmental behavior

Environmental behaviors can be divided into general behaviors and particular behaviors. The highest mean score was 5.79 for the general behavior “GB2: I will read reports or books about the local environment” and the second highest mean score was 5.67 for the particular behavior “PB3: I will pick up trash and branches when I see them on the beach.” The lowest mean was 5.52 for the question “PB1: When I see others causing damage to the local environment, I will bring it to the attention of the relevant authorities,” which is also a particular behavior. The results show that students are proactive in learning about environmental issues, but they may not have the courage to report any environmental damage in real life or they may not know how to report it.

### Measurement model analysis

Before performing the overall PLS model analysis, the reliability of all questions and components should be analyzed to ensure the reliability of the hypothesis validation. In this study, SPSS 26.0 was used to examine the reliability of each component. As shown in Table 1, the Cronbach’s alpha for science learning self-efficacy was 0.92, the alpha for environmental attitudes was 0.95, and the alpha for environmental behaviors was 0.90. The alpha of each component is greater than 0.90 (Vaske et al., 2017), which means that this questionnaire has good internal consistency. However, the requirements or special limitations of each study, some scholars have proposed different criteria, for example, the validity analysis (CFA) factor loadings above 0.5 can keep the question (Hair et al., 2006). Table 2 also shows that the factor loadings of all questions are greater than 0.5, which means that the questions can explain the components effectively (Fornell and Larcker, 1981; Shrestha, 2021).

**TABLE 2:** The convergent validity and discriminant validity of the measurement model.

Factors and items	M	SD	CR	AVE	EA	EB	SSLSE
EA	5.57	1.16	0.92	0.80	**0.90** ^a^		
EB	5.62	0.87	0.88	0.79	0.84	**0.89** ^a^	
SSLSE	5.70	0.93	0.93	0.77	0.67	0.65	**0.88** ^a^

M, mean; SD, standard deviation; CR, construct reliability; AVE, average variance extracted.

^a^Bold numbers on the diagonal parentheses are the square root of each construct’s AVE; *p* < 0.001.

In terms of measuring the validity of the model, if the average variances extracted (AVE) exceeds 0.50, it means that the components have good convergent validity (Fornell and Larcker, 1981; dos Santos and Cirillo, 2021). As shown in Table 2, the average variance extractions of the components ranged from 0.77 to 0.80, indicating good convergent validity among the components. In addition, the square root of AVE is larger than the number of correlation coefficients of each component which accounts for more than 75% of the overall comparisons, indicating that the components have discriminant validity (Hair et al., 1998; Mundfrom et al., 2005).

### Structural model analysis

**FIGURE 4:**
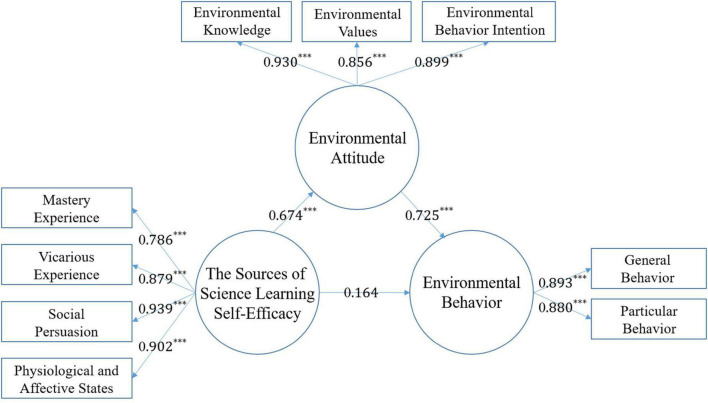
The validated proposed model. *p < 0.05 (significant); **p < 0.01 (highly significant); ***p < 0.001 (extremely significant).

The validation results of the hypothesis model in this study are shown in Figure 4 and Table 3. The structural model path coefficient is 0.725 (*t* = 6.372, *p* < 0.001) for hypothesis 1 that EA positively affects EB; 0.164 (*t* = 1.079, *p* > 0.05) for hypothesis 2 that SSLSE positively affects EB; and 0.674 (*t* = 5.674, *p* > 0.05) for hypothesis 3 that SSLSE positively affects EA; 0.674 (*t* = 5.901, *p* < 0.001). In summary, hypothesis 1 and 3 are valid, meaning that SSLSE positively affects EA and EA positively affects EB, while hypothesis 2 is not valid, meaning that SSLSE does not positively affect EB.

**TABLE 3:** Examination of hypothesized pathways.

Hypotheses	Original sample (O)	*t*	*p*	Established or not established
H1: EA → EB	0.725	6.372***	<0.001	Established
H2: SSLSE → EB	0.164	1.079	0.281	Not established
H3: SSLSE → EA	0.674	5.901***	<0.001	Established

*p < 0.05 (significant), **p < 0.01 (extremely significant), and ***p < 0.001 (extremely significant).

As a result of the aforementioned findings, (H1: EA → EB) students were more willing to engage in environmental protection behaviors and try to discuss local environmental protection issues with others (environmental behavior intention) when they were more knowledgeable about the local environment (environmental knowledge is one of the components in the environmental attitude scale), believed that the natural environment must be protected, and read more reports or books about the local environment (understanding environmental values). Although (H2: SSLSE → EB) the AR digital picture book can be used in environmental education courses to enhance students’ understanding and appreciation of the local scenery, and to gain satisfaction and improvement from the experience, it cannot directly drive students to engage in environmental protection-related activities in their daily lives. The reason for this is that the students in this study come from different cultures. Therefore, when they find environmental damage, they do not know how to report it to the relevant authorities and feel that they cannot change the situation with their own power. However, it does not directly drive the students to engage in environmental protection-related activities in their daily lives, (H3: SSLSE → EA) but only affects their environmental attitudes. Through the AR digital picture book, environmental knowledge is enhanced, environmental values are understood and positive environmental behavior intentions of students are increased. Therefore, in this study, the mediating effect of environmental attitudes will be further investigated to verify whether the students’ science learning self-efficacy can influence environmental behavior through environmental attitudes.

### The intermediary effect of environmental attitude test

The first three conditions must be met to verify the mediating effect: first, the independent variable must significantly affect the dependent variable; second, the independent variable must significantly affect the mediating variable; third, when the mediating variable is added between the independent variable and the dependent variable, the independent variable must significantly affect the mediating variable and the mediating variable must significantly affect the dependent variable.

Finally, the coefficient of influence between the independent variable and the dependent variable was used to determine the mediating effect (Preacher and Hayes, 2008). In this study, the independent variable was science learning self-efficacy, the dependent variable was environmental behavior, and the mediating variable was environmental attitude (Hypothesis 4). For the validation condition 1, the results of the survey analysis indicated that science learning self-efficacy positively influenced environmental behaviors with a standardized path coefficient of 0.489 (*t* = 3.14, *p* < 0.001, *R*^2^ = 0.36). For Condition 2, the analysis revealed that science learning self-efficacy positively influenced environmental attitudes by 0.674 (*t* = 5.85, *p* < 0.001, *R*^2^ = 0.455). For Condition 3, when environmental attitudes were between science learning self-efficacy and environmental behaviors, science learning self-efficacy positively influenced environmental attitudes and environmental attitudes positively influenced environmental behaviors. In addition, the standardized path coefficient between scientific learning self-efficacy and environmental behavior was found to decrease by 0.164 (*t* = l.04, *p* > 0.05, *R*^2^ = 0.713) compared to that without the inclusion of the mediating variable. From the above analysis, the coefficient of the pathway between science learning self-efficacy and environmental behavior decreased from a significant positive effect (0.489) to a non-significant positive effect (0.164) after the inclusion of the mediating variable, but the overall explanatory power increased from 0.489 to 0.653. According to the above results, science learning self-efficacy positively influences environmental behaviors under the original model test, but positively influences environmental behaviors through environmental attitudes, resulting in an increase in the explanatory power of environmental behaviors (Yzerbyt et al., 2018).

### Discussion of the results of qualitative data analysis

Based on the use of AR digital picture books in this research experiment and the qualitative data from some students’ interviews, a summary of the impact of AR picture books on the teaching outcomes of the classroom is presented in the following list.

(1) AR digital picture books are easily disturbed by the environment, such as the angle of the camera and the lack of light source, which may cause errors in recognition. In addition, the interface design of the digital picture book is not user-friendly enough, especially for students with different cultural backgrounds, and the graphic interface can be added in the future to facilitate learning activities.
(2) Although AR digital picture books are fun to overlay with the real environment, the interactive mode is simple and will make students feel tired after a few repetitions, which will easily lead to low reading fidelity. Moreover, some students are weak in spatial thinking, so the use of AR digital picture books for environmental education courses will cause a burden to them.
(3) The future design of AR digital picture books is desirable to reduce the limitations of using carriers in order to enhance the effectiveness of cross-curricular learning. However, the limitations and effectiveness of its use in education and its challenges and functions on the educational environment need to be researched on an ongoing basis.
(4) For Japanese students, the aboriginal culture of Taiwan is very foreign to them, but with the help of AR technology, they can be educated on cross-cultural communication and understanding. This also means that the key to AR technology application is not in the awesome visual effects, but in the real meaning of linking virtual and real-life data, objects and experiences to find new ways of learning.
(5) After the class, some of the participants said that the AR digital picture book teaching had increased their desire to learn more after the class, and they would want to be more active in finding new applications or showing others around them about the tool. This has also increased the interaction between parents and children, who will discuss and find answers with parents, and even find out if there are other AR digital picture books or apps to compare and share.

Integrating the statistical scales and the above-mentioned experiences of teachers and students, it was verified that the AR digital book can effectively enhance environmental attitudes through visual knowledge presentation, but cannot directly influence environmental behaviors. It is hypothesized that because students have an adjustment period when learning new technologies, it would be helpful to add simple explanations, guidelines, and more interactive learning content when using AR digital books for the first time. This will make the system more straightforward to use and lower the barrier to entry for users, so that the tool can be applied more easily and flexibly.

## Discussion and future prospects

In addition to validating the traditional theories of environmental attitudes and behaviors, this study also included the factor of science learning self-efficacy gained from AR digital picture book environmental education in order to understand whether AR digital picture books could influence students’ environmental behaviors. The research results suggest that when students’ environmental knowledge, environmental values, and environmental behaviors increase, they are more likely to engage in responsible environmental behaviors, which is similar to the findings of Kaiser et al. (1999). Regarding the issue that enhancing students’ knowledge about the environment affects their environmental behaviors, the findings suggest that when students are more aware of their surroundings, they are more willing to engage in environmentally friendly behaviors (Kim et al., 2018). We have also found that value change affects perceptions of environmental issues, which in turn affects students’ environmental behaviors, such as greater emphasis on recycling, waste separation, and use of renewable energy in daily life (Steg and Vlek, 2009). Therefore, behavioral intention is an important predictor of behavior, and when environmental-related policies or activities are actively promoted through various multimedia, students may be motivated to actually engage in environmentally beneficial behaviors in their daily lives (Huang, 2016).

On the other hand, the results of this study did not find a direct effect of science learning self-efficacy on environmental behaviors, although the results differ from Zareie and Navimipour’s (2016) study that indicated a direct effect of electronic environmental knowledge learning (ELE) on students’ environmental behaviors through good electronic environmental knowledge learning. The reason for this may be that the AR digital picture book contains the Taiwanese Dao indigenous culture, which may be difficult for Japanese elementary school students to understand. This means that when students read the contents of AR digital picture books and learn about local cultures and environmental issues, the emotional satisfaction they receive may enhance their positive attitudes toward the local environment and lead to support related conservation policies and activities in their daily lives. Therefore, it is important to enhance environmental literacy and self-efficacy of environmental education teachers in order to more easily inspire students’ understanding of environmental issues across cultures (Saribas et al., 2014, 2017). Finally, the limitations and recommendations of the study are that the sample size and sample characteristics are relatively small and tend to be homogeneous, and the sample size should be increased in the future to improve the reliability of the study. Furthermore, the AR digital picture book environmental education content can be diversified to avoid restricting to a single topic so as to understand whether science learning self-efficacy, environmental attitudes and environmental behaviors have similar patterns of influence in different natural environments. In conclusion, this study did not specifically examine the changes in science learning self-efficacy, environmental attitudes, and environmental behaviors before and after the AR digital picture book environmental education. A comparison of research models before and after the implementation of AR digital picture book environmental education may be warranted in future research.

## Data availability statement

The original contributions presented in this study are included in the article/supplementary material, further inquiries can be directed to the corresponding author.

## Ethics statement

The studies involving human participants were reviewed and approved by the National Cheng Kung University Governance Framework for Human Research Ethics. Written informed consent to participate in this study was provided by the participants’ legal guardian/next of kin.

## Author contributions

The author confirms being the sole contributor of this work and has approved it for publication.
